# Supplementary material for: Comparison of Various Extraction Approaches for Optimized Preparation of Intracellular Metabolites from Human Mesenchymal Stem Cells and Fibroblasts for NMR-Based Study
Source: Metabolites. 2024 May 7;14(5):268. doi: 10.3390/metabo14050268 (PMC11122815; doi:10.3390/metabo14050268)

Comparison of various extraction approaches for optimized preparation of intracellular metabolites from human mesenchymal stem cells and fibroblasts for NMR-based study.

Slavomíra Nováková[1], Zuzana Hatoková[1], Gábor Beke[2], Janka Pálešová[1], Romana Záhumenská[1], Bibiána Baďurová[1], Mária Janíčková[3], Ján Strnádel[1], Erika Halašová[1], Eva Baranovičová[1], Henrieta Škovierová[1]*

[1]Biomedical Centre Martin, Jessenius Faculty of Medicine in Martin, Comenius University in Bratislava (JFM CU), Malá Hora 4C, 036 01 Martin, Slovakia.

[2]Institute of Molecular Biology, Slovak Academy of Sciences, Dúbravská cesta 21, 845 51 Bratislava, Slovakia

[3]Department of Stomatology and Maxillofacial Surgery, University Hospital in Martin and JFM CU, Kollárova

Author for correspondence: eva.baranovicova@uniba.sk, Tel.: + 421432633495

Table of content

Supplementary figure S1. Morphology of DPSCs and HDFa cells at four days after incubation when they were applied in experiments , which were targeted to effect of harvesting (A) or extraction (B) methods. Magnification 100 x

A/. Effect of harvesting method

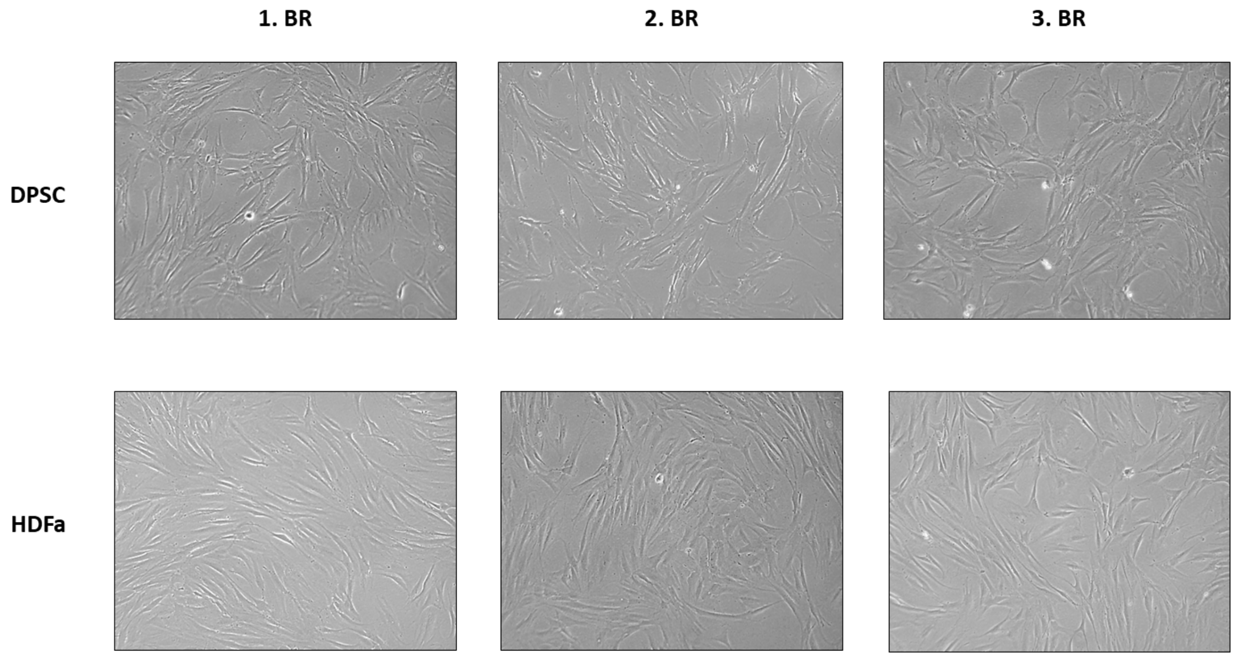

B/. Effect of extraction method

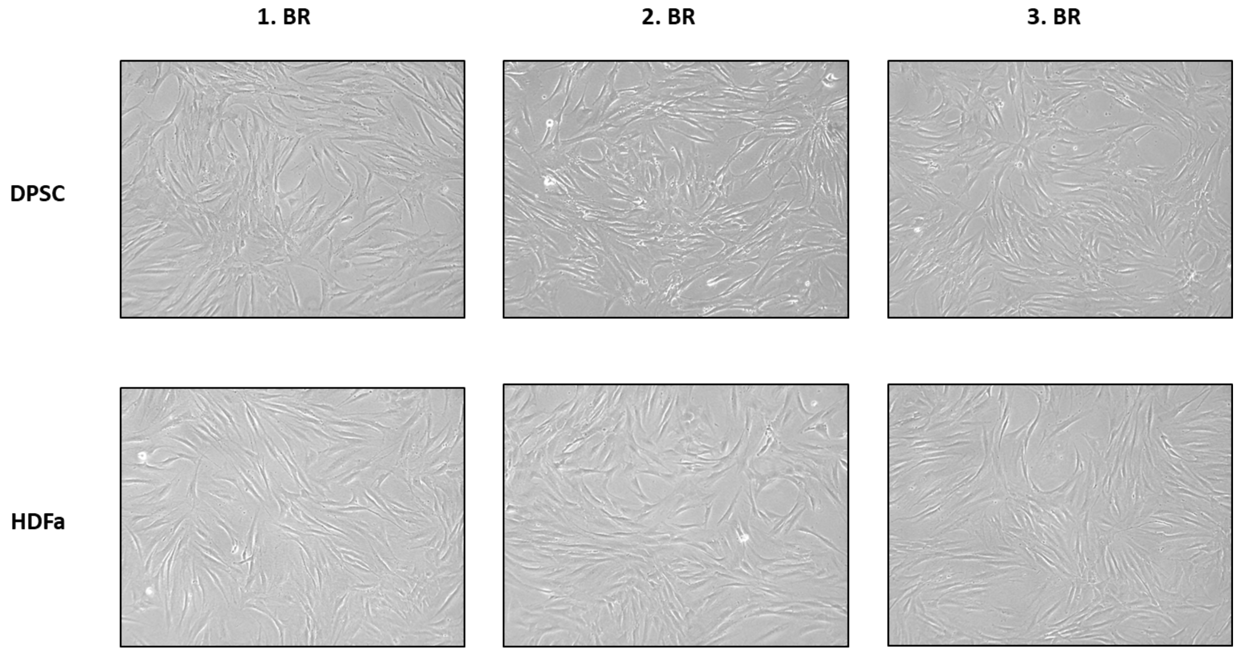

Supplement: Supplementary file 1 [file metabolites-14-00268-s001.zip › Supplementary figures, Figure S1.pdf]
